# Deglacial Tropical Atlantic subsurface warming links ocean circulation variability to the West African Monsoon

**DOI:** 10.1038/s41598-017-15637-6

**Published:** 2017-11-13

**Authors:** Matthew W. Schmidt, Ping Chang, Andrew O. Parker, Link Ji, Feng He

**Affiliations:** 1 Department of Ocean, Earth and Atmospheric Sciences, Old Dominion University, Norfolk, VA 23529 USA; 2 Department of Oceanography, Texas A&M University, College Station, TX 77843 USA; 3 Center for Climatic Research, Nelson Institute for Environmental Studies, University of Wisconsin-Madison, Madison, WI 53706 USA; 4 College of Earth Ocean and Atmospheric Sciences, Oregon State University, Corvallis, Oregon, 97331 USA

## Abstract

Multiple lines of evidence show that cold stadials in the North Atlantic were accompanied by both reductions in Atlantic Meridional Overturning Circulation (AMOC) and collapses of the West African Monsoon (WAM). Although records of terrestrial change identify abrupt WAM variability across the deglaciation, few studies show how ocean temperatures evolved across the deglaciation. To identify the mechanism linking AMOC to the WAM, we generated a new record of subsurface temperature variability over the last 21 kyr based on Mg/Ca ratios in a sub-thermocline dwelling planktonic foraminifera in an Eastern Equatorial Atlantic (EEA) sediment core from the Niger Delta. Our subsurface temperature record shows abrupt subsurface warming during both the Younger Dryas (YD) and Heinrich Event 1. We also conducted a new transient coupled ocean-atmosphere model simulation across the YD that better resolves the western boundary current dynamics and find a strong negative correlation between AMOC strength and EEA subsurface temperatures caused by changes in ocean circulation and rainfall responses that are consistent with the observed WAM change. Our combined proxy and modeling results provide the first evidence that an oceanic teleconnection between AMOC strength and subsurface temperature in the EEA impacted the intensity of the WAM on millennial time scales.

## Introduction

Recent droughts across West Africa resulted in catastrophic socio-economic impacts on the developing nations in the region and highlight the need for a better understanding of the causes for these events^1^. Studies show that WAM variability is linked to changes in the land-sea temperature contrast that is essential for monsoon development^2–6^. Furthermore, modern observational data and modeling studies show that a slowdown in the AMOC results in a subsurface warming in the tropical Atlantic, which, in turn, can impact the land-sea temperature contrast via upwelling and therefore impact the strength of the WAM refs^7,8^. If confirmed, this mechanism would explain an important oceanic teleconnection linking ocean circulation patterns in the high-latitude North Atlantic to tropical climate dynamics that could abruptly affect the intensity of the WAM.

Under modern climate conditions, warm salinity maximum waters of the subtropical North Atlantic gyre are isolated from cooler, fresher subsurface waters in the Tropical Atlantic by the strong northward return flow of the AMOC along the western boundary (North Brazil Current (NBC)) refs^9–12^. Modeling results show that a reduction in AMOC allows the warm subsurface waters of the North Atlantic subtropical gyre to flow south and warm the subsurface along the western boundary^8^. Modeling results from ref.^13^ showed that a similar reduction in AMOC under Last Glacial Maximum (LGM) boundary conditions also resulted in a subsurface warming across the tropical Atlantic, suggesting this oceanic mechanism operated in the past. In these modeling experiments, the subsurface warming is propagated into the EEA where it impacts sea surface temperatures (SSTs) in the EEA cold tongue through equatorial and coastal upwelling. The mechanism linking subsurface to surface temperature response is through reduction in the vertical temperature gradient which reduces upwelling-induced cooling. The model simulations find that a warming of SSTs in the EEA cold tongue results in a reduced land-sea temperature gradient, which in turn weakens the strength of the WAM.

However, up to now, there is lack of supporting observational evidence of thermocline temperature change in the EEA to validate these modeling results. Although ref.^13^ provided proxy evidence for a warming in the western Tropical Atlantic during periods of reduced AMOC across the deglaciation, results from the southern Caribbean do not provide direct evidence for the oceanic teleconnection linking variability in the AMOC to changes in the WAM system. In order to fully test this mechanism, we present new proxy evidence from thermocline depths in the EEA and a new transient coupled ocean-atmosphere model simulation across the YD. We then use our new thermocline temperature record to validate the transient modeling results.

**Figure 1:**
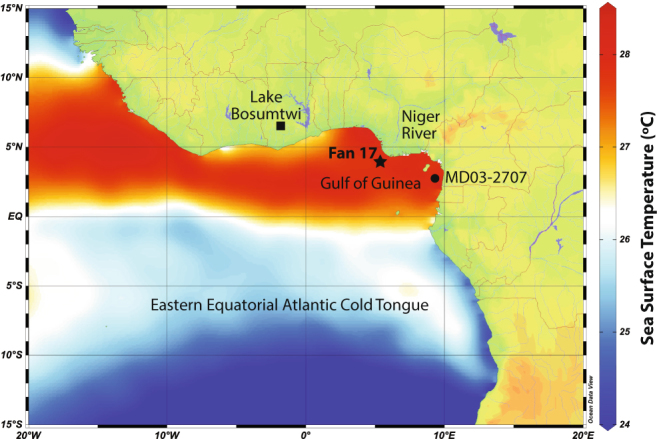
Regional map of the Eastern Equatorial Atlantic showing locations of Fan 17 (star) and the nearby core locations of MD03-2707 (circle) (ref.^33^) and Lake Bosumtwi^48^ (square). Colors represent mean annual SST in °C from ref.^17^. Cooler SSTs south of the equator outline the EEA cold tongue. The authors wish to acknowledge use of Ocean Data View (*Schlitzer, R., Ocean Data View*, http://odv.awi.de *, 2015*) to make this figure.

Sediment core Fan 17 (4.81°N, 4.45°E, 1178 m) was recovered from the EEA equidistant (~100 km SW) between the two major distributaries of the Niger River Delta (Fig. 1). The radiocarbon based deglacial age model for Fan 17 was previously published^14^ (see Methods for more details). Freshwater discharge at the site is sourced entirely from runoff of the Niger River, and the magnitude of this discharge has been shown to directly correlate with the strength of the WAM (ref.^15^ (Fig. SI 1). Here, we use Mg/Ca ratios in the thermocline dwelling planktonic foraminifera *Globorotalia crassaformis*, (modern depth habitat between 200–270 m in the EEA ref.^16^), as a proxy for thermocline temperature variability.

## Results

### Subsurface Temperature Reconstruction

**Figure 2:**
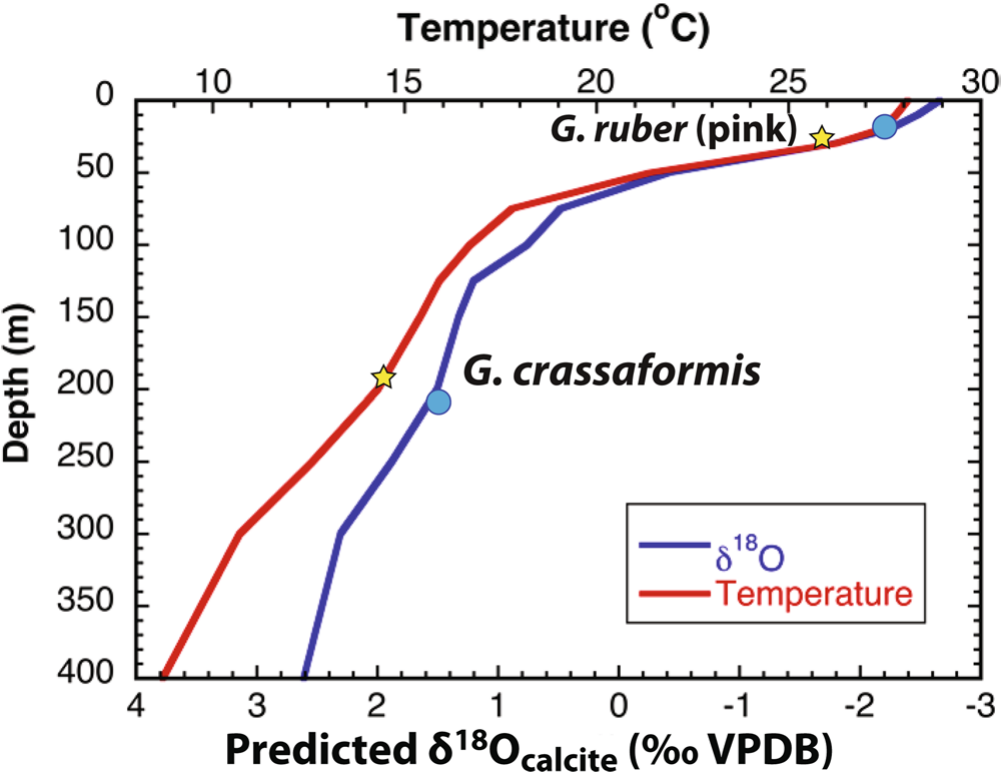
Modern thermocline at the Fan 17 location in the Niger Delta (red line) was constructing using WOCE data^17^. Predicated δ^18^O (blue line) calculated following the steps outlined in ref.^49^ using annual hydrographic data to convert to δ^18^O_seawater_ and then solving the δ^18^O-temperature equation of ref.^50^ for δ^18^O_calcite_. Plotted are the core top Mg/Ca temperatures for *G. ruber* and *G. crassaformis* (yellow stars) as well as core top δ^18^O_calcite_ values for both species (blue circles). Core top SST for *G. ruber* is consistent with a habitat depth in the upper most mixed layer while *G. crassaformis* temperature is consistent with a habitat in the lower thermocline. For both species, the δ^18^O_calcite_ values are consistent with core top temperature estimates and habitat depths.

To better constrain the depth habitat of *G. crassaformis* in the Niger Delta, we measured δ^18^O values and Mg/Ca ratios of specimens from the core-top interval with an age of ~550 years. We then calculated the predicted shell δ^18^O value vs. depth profile based on modern temperature and salinity data^17,18^. Comparison of our *G. crassaformis* δ^18^O core-top values with the predicted δ^18^O values places the depth habitat of this species at ~225 m in the Niger Delta region (Fig. 2). In addition, the calculated core-top Mg/Ca temperature for *G. crassaformis* suggests its modern habitat depth is ~200 m, giving us confidence that the depth habitat of *G. crassaformis* at our study site is consistent with the previously documented depth preference for this species in upwelling systems^16^. Although deep dwelling planktonic foraminifera can migrate vertically in the water column through time^19,20^, a deeper depth habitat for *G. crassaformis* would still place it within the depth range of subsurface warming in the EEA associated with an abrupt decrease in AMOC strength based on the modeling results of our new transient YD simulation (see Fig. S2).

**Figure 3:**
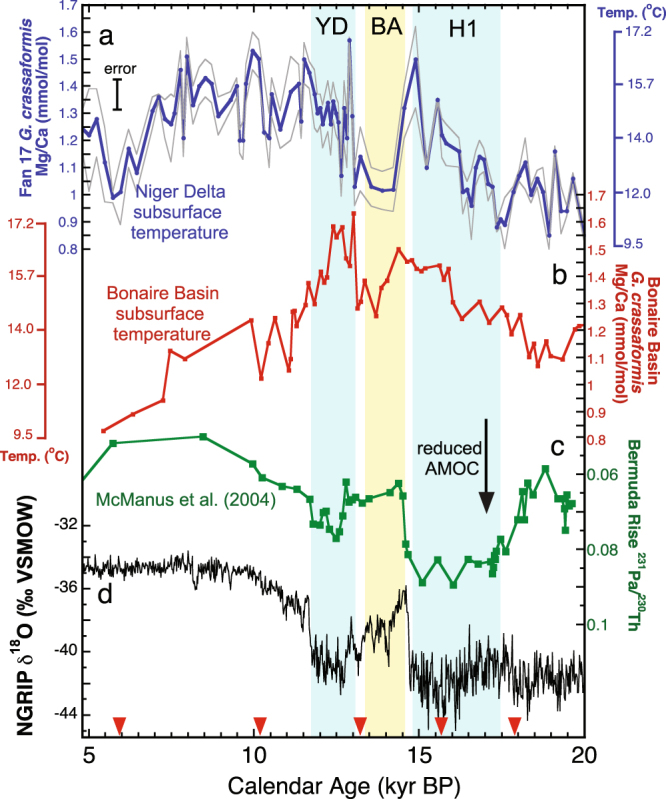
Tropical Atlantic subsurface temperature records and AMOC variability across the deglaciation. (**a**) Fan 17 *G. crassaformis* Mg/Ca ratios (blue circles) with the error plotted for all duplicate runs shown as the gray envelope. Although 80% of the intervals were run in duplicate, the pooled standard deviation calculated for all Mg/Ca measurements (+/−0.07 mmol/mol) was used to estimate error for the 20% of the intervals with only a single analysis. The corresponding temperature scale calculated using the *G. crassaformis* Mg/Ca-temperature equation from ref.^21^ is plotted along the right y-axis. (**b**) *G. crassaformis* subsurface Mg/Ca and corresponding temperature record from the Bonaire Basin in the southern Caribbean plotted as red squares^13^. (**c**) The^231^Pa/^230^Th record of AMOC variability from the Bermuda Rise^28^ and (**d**) NGRIP ice core record^51^. Red triangles along the x-axis indicate calibrated radiocarbon dates. Blue bars note H1 and the YD and the yellow bar marks the Bølling Allerød warm interval.

Acknowledging there is debate about the Mg/Ca-temperature calibration for deep-dwelling planktonic foraminifera, we focus on relative changes in *G. crassaformis* Mg/Ca ratios as a proxy for subsurface temperature variability across the deglaciation. Mg/Ca ratios in *G. crassaformis* increase from 0.9 to 1.1 mmol/mol at the start of H1 at 17.5 kyr (equating to a >20% increase in shell Mg/Ca at this time) (Fig. 3a). Overall, shell Mg/Ca ratios continue to increase through the rest of H1, reaching a maximum Mg/Ca ratio of 1.5 mmol/mol at 15 kyr. This equates to a total Mg/Ca increase of 0.5 mmol/mol across the entire cold event, well above the pooled standard deviation on our replicated Mg/Ca measurements of ±0.07 mmol/mol (see methods). Based on one of the species-specific Mg/Ca-temperature calibrations for *G. crassaformis* ^21^, the total warming over the period from 17.5-15 kyr suggests a subsurface temperature increase of up to ~5 °C. Immediately following H1, *G. crassaformis* Mg/Ca ratios decrease by 0.5 mmol/mol, indicating cooler subsurface conditions in the Niger Delta at the start of the Bølling-Allerød (BA) interstadial beginning at 14.8 kyr. Cooler subsurface conditions persist until another abrupt increase of 0.3 mmol/mol in Mg/Ca ratios occurs at the onset of the YD at 13 kyr (Fig. 3a). Therefore, shell Mg/Ca ratios increase by more than 20% over BA values at the start of the YD, well above the estimated error of 6% on our Mg/Ca measurements.

### YD Transient Simulation

**Figure 4:**
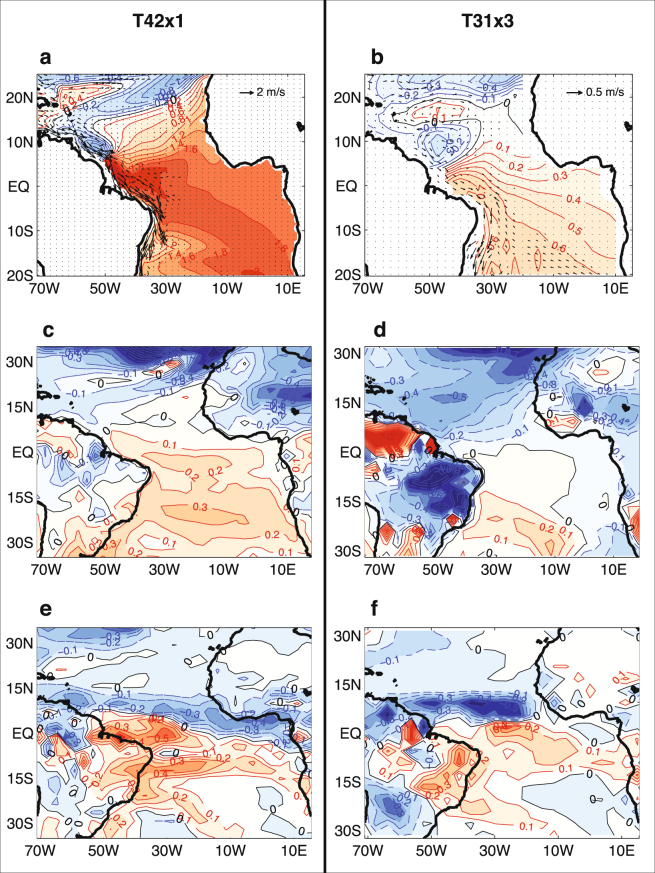
Comparison of high- vs. low- resolution CCSM3 YD transient simulations. All the results shown are based on averages during mid-YD from 12.1 to 12.3 kyr relative to 12.85 kyr. (**a**) and (**b**) Changes in temperature (color and contour) and velocity (vector) averaged between 200 and 275 m in the T42x1 CCSM3 simulation (this study) and in the T31x3 CCSM3 simulation^23^, respectively. (**c**) and (**d**) Changes in surface temperature in the T42x1 CCSM3 simulation and in the T31x3 CCSM3 simulation, respectively. (**e**) and (**f**) Same as in (**c**) and (**d**) except for changes in precipitation. The authors wish to acknowledge use of the Matlab (https://www.mathworks.com/products/matlab.html) for this graphic.

Previous modeling studies conducted a transient climate simulation of the last deglaciation using a low-resolution version of Community Climate System Model Version 3 (CCSM3) (TraCE, refs^22,23^. The CCSM3 model is comprised of a T31 spectral truncation (approximately 3.75° horizontal resolution) Community Atmosphere Model 3 (CAM3) with 26 hybrid coordinate levels in the vertical and a 3°x3° Parallel Ocean Program (POP) with 25 vertical z-coordinate levels (T31x3). An examination of the YD simulation in TraCE (hereafter referred to as T31x3 YD simulation), (Fig. 4b) reveals no significant subsurface warming in the western Tropical North Atlantic and Southern Caribbean region during the YD, which is at odds with previously published proxy and GCM modeling evidence in refs^8,13^. We argue that this discrepancy is partially attributable to the coarse resolution of the ocean model used by ref.^23^, which does not properly resolve western boundary current dynamics and thus does not accurately represent the regional ocean circulation changes that are crucially important for the subsurface warming mechanism outlined in refs^8,13^.

To remedy this problem, we conducted a 1,400 year transient simulation for the YD using a higher resolution version of CCSM3 that consists of a T42 spectral truncation (approximately 2.8° horizontal resolution) CAM3 with 25 vertical levels and 1°x1° POP with 40 vertical levels (hereafter referred to as T42x1 YD simulation) (Fig. 4a). This version of CCSM3 was used by ref.^24^ to conduct a set of freshwater hosing experiments under LGM forcings and boundary conditions. The results show a major reduction in Florida Straits transport of ~10 Sv accompanied by an AMOC reduction of ~11 Sv, which is consistent with the reconstructed Florida Current transport across the Florida Straits during H1 and the YD (ref.^26^). A recent study by ref.^26^ showed that when forced with observationally based atmospheric surface fluxes, the model reproduces the observed mean AMOC depth profile at 26.5°N derived from the 4-year mean RAPID data. Collectively, these previous studies give us confidence that the 1°x1° POP is able to significantly improve western boundary current dynamics and the AMOC over the low-resolution counterpart, making it more suitable for studying the oceanic teleconnection mechanism described by refs^8,13^ under past climate conditions.

**Figure 5:**
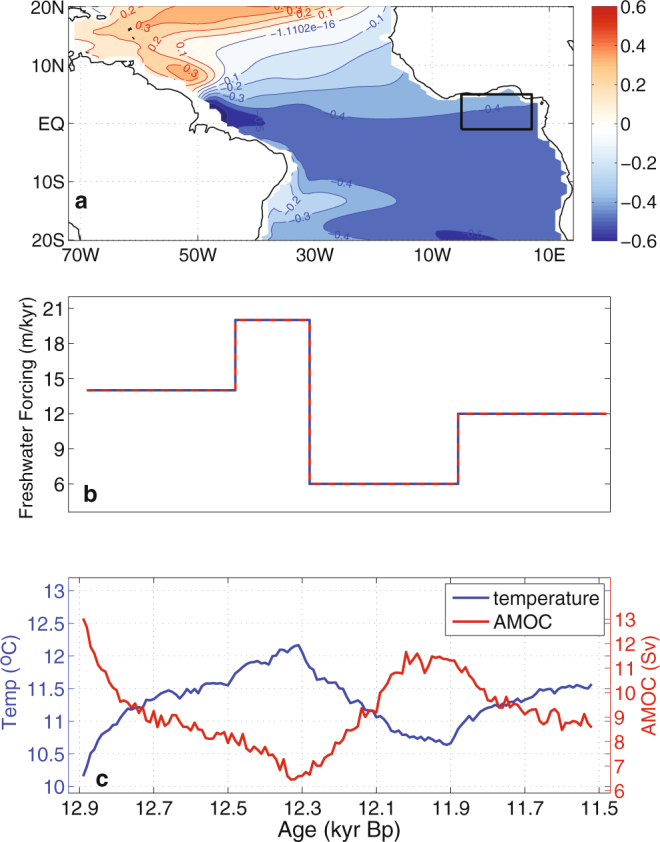
High-resolution CCSM3 YD transient simulation averaged between 200–270 m water depth. (**a**) Regression map of simulated ten-year mean temperature against a ten-year mean AMOC index defined by ten-year mean maximum volume transport streamfunction between 0°–60°N and 500–5000 m (in °C/Sv). Negative regression values indicate a warming response to weakening AMOC. Black box indicates the area over which the averaged time series in (**c**) was derived. (**b**) Freshwater forcing of the YD transient model simulation, based on a previously published sea level rise reconstruction^52^. (**c**) Ten-year mean subsurface temperature time series (blue) and AMOC index (red) for the entire YD simulation period between 12.9 and 11.6 kyr BP for the Niger Delta region. The correlation between the AMOC index and Niger Delta subsurface temperature time series is −0.96, which is significant at 95% level based on a two-sided student’s t-test. The authors wish to acknowledge use of Matlab (https://www.mathworks.com/products/matlab.html) for this graphic.

At the start of the T42x1 YD simulation at 12.9 kyr, the simulated AMOC has a maximum of ~13 Sv (Fig. 5c). This value is considerably higher than the corresponding value T31x3 YD simulation by ref.^23^, which was around 8 Sv. The AMOC strength in T42x1 YD simulation then decreases to a minimum value of ~6.5 Sv at 12.3 kyr, compared to the lowest value of ~4.5 Sv in the T31x3 YD simulation. Consistent with the stronger change in the AMOC, the T42x1 CCSM3 also produces more extensive surface cooling in the North Atlantic sector than T31x3 CCSM3 run, which causes land surface cooling over West Africa (Fig. 4c and d). Over the equatorial and south tropical Atlantic, the T42x1 YD transient run further produces a surface warming of ~0.2–0.3 °C averaged over the mid-YD period between 12.3 and 12.1 kyr (in reference to 12.85 kyr), while in the T31x3 YD simulation no such surface warming was observed (Fig. 4c and d). The combined effect of the land surface cooling and ocean surface warming is to reduce the land-ocean thermal contrast over the WAM region. As a result, there is a well-defined southward shift of the monsoon rainfall towards to the warmer ocean in the T42x1 YD transient run, which is consistent with a reduction in WAM. (Fig. 4e). In contrast, the rainfall response over the West Africa and Gulf of Guinea region is much weaker in the T31x3 YD simulation (Fig. 4f), presumably due to the lack of the surface warming in the equatorial south tropical Atlantic.

This lack of the surface warming can be attributed to the striking difference between the two simulations occurs in the subsurface temperature response. Clearly, the T42x1 YD simulation produces a much more pronounced subsurface temperature response to AMOC changes than the T31x3 YD run (Fig. 4a and b). In the T42x1 YD run, a strong subsurface warming (averaged between 200 m and 275 m) of ~3 °C is found along the western boundary near the gyre boundary of the tropical and northern subtropical gyres in the mid-YD (Fig. 4b). Accompanying the warming is a strong reduction in the NBC and a broad subsurface warming of 1.6 °C–1.8 °C along the equatorial waveguide. This subsurface response has been explained by a two-stage process in refs^8,13^. The first stage is a wave adjustment process in which warm Kelvin waves bring the subsurface warming signal from high-latitude North Atlantic to the equatorial Atlantic^27^ and the second stage is a western boundary current response in which the weakened AMOC returned flow causes the North Brazil Current (NBC) to weaken, allowing the warm salinity maximum waters (SMW) of the subtropical North Atlantic gyre to enter the equatorial Atlantic^9^. In contrast, the T31x3 YD run shows no evidence of NBC change and strong subsurface warming along the western boundary and the equatorial warming (~0.3 °C) is considerably weaker than that in the T42x1 YD run. These findings point to the importance of resolving western boundary current dynamics in simulating AMOC-induced tropical climate response and strongly suggest that the T42x1 CCSM3 is more suitable for simulating the tropical response to AMOC slowdowns.

## Discussion

The goal of our T42x1 YD simulation spanning over 1,400 years from 12.9 to 11.5 kyr is to identify the possible mechanism responsible for abrupt subsurface warming in the tropical Atlantic. Based on the results, a regression analysis of temperature change between 200–270 m (the depth habitat of *G. crassaformis* in the EEA) against the AMOC index (defined by the maximum value of the overturning streamfunction in the North Atlantic) shows that under YD forcings and boundary conditions, subsurface temperatures south of 10°N vary in opposite phase to AMOC (Fig. 5a). In other words, a weakened AMOC leads to an increase in thermocline temperatures in the EEA. The strongest surface temperature response occurs along the NBC and equatorial waveguide, as expected from the oceanic teleconnection mechanism^8,13^. Along the Guinea coast, the regression coefficient has a value of −0.4 °C/Sv (see black box on Fig. 5a).

To compare directly with our new temperature record, we derived a subsurface temperature time series for our study area by averaging the simulated temperature from 200–270 m over the region indicated by the rectangle in Fig. 5a. Figure 5c shows the temperature time series (blue) along with the simulated AMOC index (red) during the entire transient simulation period. Results show that subsurface temperatures in the EEA and AMOC strength are anti-correlated. As the AMOC strength decreases rapidly in the early stage of the YD from 12.9 kyr to 12.3 kyr, the subsurface temperature undergoes a rapid warming of ~2 °C and reaches a maximum value around 12.3 kyr. Then, as AMOC begins to strengthen between 12.3–12.0 kyr due to reduced freshwater input into the North Atlantic in the model, subsurface temperatures in the EEA cool by 1.5 °C.

Several records showed that^231^Pa/^230^Th ratios at core sites in the North Atlantic approach near-production levels during H1 and the YD, suggesting that AMOC was significantly reduced during these two cold intervals (Fig. 3c) (refs^28,29^). As shown on Fig. 3a, these are also times of increased subsurface temperatures in the Niger Delta based on our *G. crassaformis* Mg/Ca record. In addition, a previously published subsurface temperature record also based on Mg/Ca ratios in *G. crassaformis* from the southern Caribbean^13^ found a significant subsurface warming during both H1 and the YD (Fig. 3b), suggesting subsurface temperatures increased in the western Tropical Atlantic region when AMOC is reduced. Taken together, these records suggest the entire thermocline of the tropical Atlantic warmed during H1 and the YD, providing important observational data supporting our new T42x1 YD simulation results.

A recent study from the Gulf of Guinea also found a subsurface warming during H1 and the YD at a depth of 1,295 m based on Mg/Ca paleothermometry in benthic foraminifera^30^. Based on a coarse-resolution transient simulation in this study, these authors invoked a 1-D downward diffusion mechanism associated with AMOC weakening to explain the intermediate depth warming in the EEA. However, the downward diffusion mechanism described in ref.^30^ is inconsistent with our results in several aspects. First, our T42x1 simulation shows that the subsurface warming in the EEA occurs at a much faster time scale than diffusion time scales. Second, the subsurface warming propagates horizontally, rather than vertically, along the western boundary from extratropical North Atlantic to the equatorial region. Taking these together, we submit that the oceanic teleconnection mechanism, which combines a fast wave adjustment and upper ocean circulation change along the low-latitude western boundary, is more in line with the high-resolution modeling results and our new thermocline temperature record.

Based on our T42x1 simulation and *G. crassaformis* Mg/Ca record, we conclude that thermocline temperatures in the tropical Atlantic rapidly respond to changes in AMOC. This finding has important implications for abrupt changes in the WAM. The northward limit of the WAM rainbelt is controlled by the intensity of the meridional temperature gradient set up by the hot Sahara to the north and the cooler SSTs in the EEA cold tongue near the equator. To this extent, Northern Hemisphere solar insolation plays a primary role in modulating the intensity of continental heating and has been shown to be the most significant forcing factor for the long-term intensity of rainfall across West Africa^31–33^. However, multiple studies have documented abrupt, millennial-scale changes in WAM strength over the Holocene and across the last deglaciation^31,34,35,4,36^ that are linked to changes in North Atlantic climate. While most of these studies have focused on how terrestrial climate and atmospheric circulation patterns have evolved across the last 21 kyrs, very little is known about the relationship between ocean temperatures in the EEA cold tongue and the strength of the WAM across the deglaciation.

**Figure 6:**
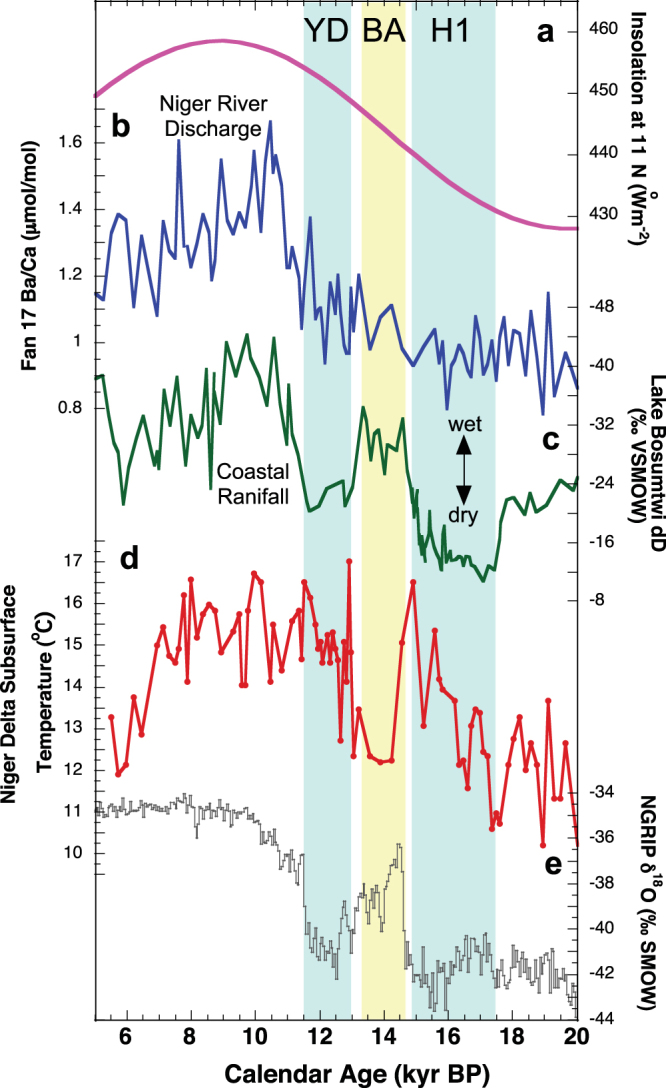
West African Monsoon variability across the deglaciation. (**a**) Northern Hemisphere summertime insolation at 11^o^N, (**b**) Fan 17 *G. ruber* Ba/Ca (μmol/mol) record reflecting changes in Niger River discharge across the deglaciation^14^ (**c**) Lake Bosumtwi δD_leaf wax_ (‰ VSMOW) (ref.^36^) (**d**), Fan 17 subsurface temperature record and (**e**) NGRIP ice core record^51^. Note that precipitation changes recorded in the Lake Bosumtwi δD_leaf wax_ record correspond to subsurface warming in the Niger Delta, most likely resulting from increased SSTs in the EEA cold tongue and reduced precipitation over land.

Next, we compare our *G. crassaformis* subsurface temperature reconstruction with the previously published record of Niger Delta discharge based on Ba/Ca ratios in the surface dwelling planktonic foraminifera *Globigerinoides ruber* (pink) from Fan 17 (ref.^14^) and shown on Fig. 6b. Although the modern Niger Delta thermocline shoals during the upwelling season, coeval with thermocline shoaling in the EEA cold tongue, it does not reach the surface due to the presence of warm, fresh waters in the delta^37^. Nevertheless, it is likely that the Fan 17 thermocline temperature record reflects regional subsurface temperature change, including in the EEA cold tongue farther to the south. Therefore, we can compare the timing of thermocline temperature change in the cold tongue to changes in the strength of the WAM.

As thermocline temperatures in Fan 17 warm across the interval from 17.5-15.0 kyr, there is no corresponding decrease in Ba/Ca ratios that would suggest decreased riverine discharge and weakening of the WAM (Fig. 6b). In fact, the Ba/Ca record suggests there is very little change in Niger River discharge from the LGM through H1. In contrast, a δD_leaf wax_ record interpreted to reflect precipitation changes in coastal Africa at Lake Bosumtwi in Ghana^36^ does record variability consistent with drier conditions in the region during H1 (Fig. 6c). During the entire period between 17.5 and 15.0 kyr when the Fan 17 subsurface record suggests warm temperatures (Fig. 6d), Lake Bosumtwi δD_leaf wax_ values become more positive, suggesting dry conditions along the Guinea Coast. During the LGM and H1, low Northern Hemisphere summertime insolation (Fig. 6a) likely limited the northward extreme of the monsoon across interior West Africa, leading to persistent low Niger River discharge. Because the rainfall had yet to intensify in the Niger River catchment area in the Sahel, the change in precipitation and thus Niger River discharge from LGM to H1 was negligible, explaining the lack of change in Ba/Ca in the Fan 17 record at this time. However, along the Guinea Coast, δD_leaf wax_ values suggest that Lake Bosumtwi (6°N) was experiencing a rainy season during the LGM. As subsurface waters began to warm the EEA cold tongue around 17.5 kyr, the weakening of the land-sea thermal gradient likely shortened the length of the rainy season over the Guinea coast as the rainbelt became biased toward the warmer waters in the cold tongue, causing a drying at Lake Bosumtwi during H1.

Subsurface temperatures then cool in the Niger Delta at the onset of the BA at 14.8 kyr, likely reducing SSTs in the EEA cold tongue. At this time, Ba/Ca ratios gradually increase in the Niger Delta record and Lake Bosumtwi δD_leaf wax_ values decrease, both reflecting an intensification of rainfall over western Africa. A potential scenario that can explain these results is that a cooling of subsurface water temperatures coupled with increased Northern Hemisphere insolation steepened the land-sea thermal contrast and as a result, the seasonal cycle of the rainbelt became more established along the African coast. This led to a dramatic increase in rainfall at Lake Bosumtwi, while to the north, the steepened thermal gradient allowed the monsoon moisture to penetrate into the southern latitudes of the Sahel. As a result, Niger River discharge increased as recorded in the *G. ruber* Ba/Ca record across the BA (Fig. 3b).

Next, an abrupt warming of subsurface waters at the start of the YD at 12.9 kyr may have once again weakened the northward progression of the monsoon, leading to conditions similar to H1. Based on the Ba/Ca record from Fan 17, precipitation remained low for the first, most intense part of the YD before increasing again at 11.9 kyr. Following the YD, peak Northern Hemisphere precessional insolation likely became the dominant forcing factor for the development of the WAM during the early Holocene, allowing the monsoon to reach its most northern limit deep into West Africa, resulting in the wettest conditions in the region at around 10 kyr (ref.^31^).

Therefore, our new T42x1 YD simulation and proxy record for thermocline temperature changes in the EEA cold tongue show that changes in the land-sea temperature gradient may be an important factor controlling the strength of the WAM on millennial time scales. When AMOC was reduced during H1 and the YD, subsurface temperatures warmed in the EEA. As these warmer subsurface conditions impacted regional SSTs, the land-sea thermal gradient would have been reduced, crippling the development of the WAM. Thus, both the gradual insolation changes and the more abrupt ocean temperature changes related to AMOC variability work in concert to control the strength of the WAM. Although pecessional insolation changes impact the long-term variability of rainfall across West Africa^32^, our new data show that oceanic influences via subsurface temperature change provide the trigger for abrupt WAM disruption at times in the past.

## Methods

TDI Brooks recovered Fan 17 in 2007 as part of a larger coring initiative for Shell International and donated the core to the Department of Oceanography at Texas A&M University for research, where it is stored in the Department’s core repository. The age model for Fan 17 was previously published in ref.^14^ and was developed using 11 radiocarbon-dated intervals of mono-specific *Globigerinoides ruber* (pink variety) specimens. Radiocarbon ages were then converted to calendar years using the CALIB 7.1 program and a standard marine reservoir age correction of 400 years^14^. Based on linear interpolation between calibrated dates, the age model shows a nearly linear sedimentation rate of 16.5 cm/kyr over the past 21 kyr.

We measured the oxygen isotope composition of *G. crassaformis* specimens from the Fan 17 core top sample to better constrain the depth habitat of this species in the Niger Delta. To limit ontogenetic and growth rate effects, 7–10 *G. crassaformis* (355–425 μm) tests per sample were sonicated in methanol and analyzed whole on a Thermo Scientific MAT 253 IR-MS with Automated Kiel IV Carbonate Device at Georgia Institute of Technology. The raw δ^18^O values were standardized using the NBS-19 standard.

Trace metal analyses were performed on ~580 μg of *G. crassaformis* calcite (7–10 *G. crassaformis* tests) from each interval. We followed the cleaning procedures of ref.^38^, which involved crushing and homogenizing the samples, methanol sonication to remove clay particulates, a reducing step to remove metal oxides, an oxidation step to remove organic material, and a weak acid leach. The samples were measured at Texas A&M on a Thermo Scientific Element XR Inductively Coupled Plasma Mass Spectrometer using isotope dilution.

Mg/Ca ratios are a well-studied and widely used tool for reconstructing past ocean temperatures. The substitution of Mg into foraminiferal calcite is endothermic and thus favored at higher temperatures^39^. Calibration studies have consistently demonstrated that temperature is the primary control of Mg/Ca ratios in foraminiferal calcite^21,40,41^. Despite a recent study that suggested salinity had a much larger effect on shell Mg/Ca ratios^42^, ref.^43^ showed that this conclusion was invalid because it was based on the incorrect application of a dissolution corrected calibration equation to calculate Mg/Ca SSTs across a wide latitudinal gradient. This highlights the importance of carefully selecting the appropriate Mg/Ca-temperature equation based on the state of calcite preservation at specific core locations^44^. At Fan 17, visual inspection of the foraminifera and the measurement of nearly constant shell weights on all of our samples suggest the shell material used in this study has not been subjected to significant dissolution. Therefore, we chose the following species-specific *G. crassaformis* equation from ref.^21^ to convert Mg/Ca ratios to calcification temperatures:

1 $$G.\,crassaformis:\mathrm{Mg}/\mathrm{Ca}=0.339\,\exp (0.09\ast {\rm{T}})({\rm{error}}\pm {0.5}^{{\rm{o}}}{\rm{C}})$$

The long-term analytical reproducibility of measured Mg/Ca ratios based on a matrix-matched standard analyzed throughout the course of this study was 1.13% for Mg/Ca. The pooled standard deviation for *G. crassaformis* sample replicates was 6.0% (df = 78 based on 99 intervals, indicating that 80% of the intervals were run in duplicate). This equates to an error on the Mg/Ca measurements of ±0.07 mmol/mol).

Our YD transient simulation begins from a T42x1 CCSM3 paleo-climate simulation forced by the climate boundary conditions of 15 kyr with a CO_2_ concentration of 214 ppmv, CH_4_ at 350 ppbv (refs^45,46^), and a reconstructed Laurentide Ice Sheet and closed Bering Strait (see ref.^47^ for detail). The 15 kyr run is chosen as the initial condition because it is sufficiently close to the onset of the YD. Initializing T42x1 CCSM3 with a restart file from the 15 kyr control run conducted by refs^22,23^, we first spin up the model for 180 years with the same climate boundary conditions used by refs^22,23^. We then changed the climate boundary conditions to those corresponding to 13 kyr, including the orbital parameter, green house gas (GHG) concentrations and ice sheets with closed Bering Strait, and integrated the model for 240 years. This run was followed by another 200-year simulation with freshwater input in Weddell Sea vicinity between 60°S–80°S and 70°W–30°E and Ross Sea vicinity between 60°S–80°S and 140°E–140°W at a rate of 1.16m/200years. Our YD transient simulation was based on these spin-up runs and integrated for 1,400 years from 12.9 kyr to 11.5 kyr with closed Bering Strait. During the transient run, the climate boundary conditions, including orbital parameters, GHG concentration, ice sheets, freshwater forcing, etc., were continuously adjusted in a manner that follows closely to the approach used by ref.^23^ in their last deglaciation transient climate simulation. In particular, a freshwater forcing was added to the high-latitude North Atlantic between 30°N–60°N and 90°W–50°W to mimic the effect of melt water discharge from the St. Lawrence River during the YD when the Laurentide Ice Sheet retreated northward out of the Great Lakes. The rate of the freshwater input varies during the simulation in accordance with ref.^23^ with a value of 14 m/kyr from 12.9 to 12.5 kyr, 25 m/kyr from 12.5 to 12.3 kyr, 6 m/kyr from 12.3 to 11.9 kyr, and 12 m/kyr from 11.9 kyr to 11.5 kyr (Fig. 5b).

## Electronic supplementary material

Supplementary Information
