# Supplementary material for: Deglacial Tropical Atlantic subsurface warming links ocean circulation variability to the West African Monsoon
Source: Sci Rep. 2017 Nov 13;7:15390. doi: 10.1038/s41598-017-15637-6 (PMC5684145; doi:10.1038/s41598-017-15637-6)
Supplement: Supplementary file 1 — Supplementary Information [file 41598_2017_15637_MOESM1_ESM.doc]

Supplementary Information:

**Deglacial Tropical Atlantic subsurface warming links ocean circulation variability to the West African Monsoon**

Matthew W. Schmidt^1^*, Ping Chang^2^, Andrew O. Parker^2^, Link Ji^2^ and Feng He^3,4^

^1^ Department of Ocean, Earth and Atmospheric Sciences, Old Dominion University, Norfolk, VA 23529.

^2^ Department of Oceanography, Texas A&M University, College Station, TX 77843.

^3^ Center for Climatic Research, Nelson Institute for Environmental Studies, University of Wisconsin-Madison, Madison, WI 53706, USA

^4^ College of Earth Ocean and Atmospheric Sciences, Oregon State University, Corvallis, Oregon 97331, USA

*Correspondence to: mwschmid@odu.edu


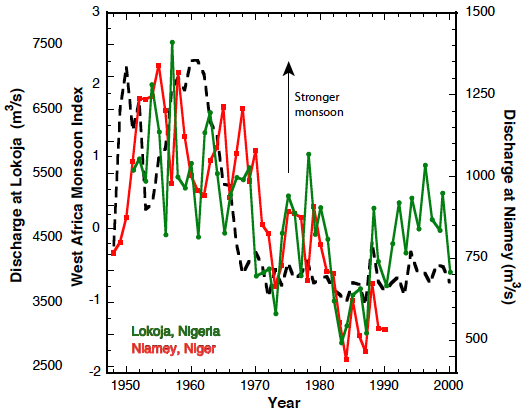


**Fig. S1:** Hydrographic data from 1950-2000 of annual upstream discharge at Niamey, Niger (red line, data from the Global River Discharge Database) and reconstructed downstream discharge from ^1^ at Lokoja, Nigeria (green line). Station locations are shown by white squares in Figure 2. Superimposed is the West African Summer Monsoon Index (black dashed line) from ^2^. Decadal weakening of the monsoon during the 1970’s is coincident with decreasing discharge at both locations.

**
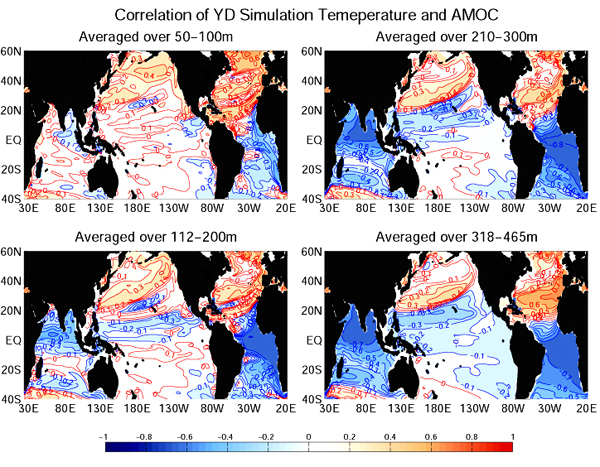
**

**
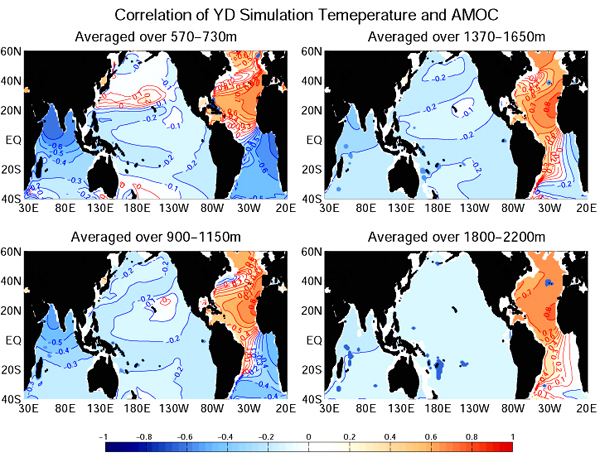
**

**Fig. S2:** Correlation coefficients between simulated AMOC index and subsurface temperature changes at different depth ranges. The negative correlation between AMOC and subsurface temperature near the study site is retained throughout the depth range between 50 m – 1500 m, so even if the depth habitat of *G. crassaformis* changed in the past, this species would still be impacted by the regional subsurface warming resulting from a reduction in AMOC. **The authors wish to acknowledge use of Matlab (https://www.mathworks.com/products/matlab.html) for this graphic.**

**References**

1 Itiveh, K. O. & Bigg, G. R. The variation of discharge entering the Niger Delta system, 1951-2000, and estimates of change under global warming. *Int J Climatol* **28**, 659-666, DOI:10.1002/Joc.1568 (2008).

2 Li, J. P. & Zeng, Q. C. A unified monsoon index. *Geophys Res Lett* **29**, DOI:10.1029/2001gl013874 (2002).
